# Categorisation of polyphonic musical signals by using modularity community detection in audio-associated visibility network

**DOI:** 10.1007/s41109-017-0052-1

**Published:** 2017-10-10

**Authors:** Dirceu de Freitas Piedade Melo, Inacio de Sousa Fadigas, Hernane Borges de Barros Pereira

**Affiliations:** 1 Department of Mathematics (DEMAT), Nucleus of Studies of Mathematics, Statistics and Education (NEMEE), Federal Institute of Education Science and Technology of Bahia (IFBA), Salvador, Bahia Brazil; 2 State University of Feira de Santana (UEFS), Feira de Santana, Bahia Brazil; 3 State University of Bahia (UNEB), Computational Modeling Program, SENAI CIMATEC, Salvador, Bahia Brazil

## Abstract

This article proposes a method to numerically characterise the homogeneity of polyphonic musical signals through community detection in audio-associated visibility networks and to detect patterns that allow the categorisation of these signals into two types of grouping based on this numerical characterization. To implement this methodology, we first calculate the variance fluctuation series in fixed-size windows of an audio stretch. Next we map this series onto a visibility graph, where the nodes are the points of the series, and the edges are defined by the visibility between each pair of points. Then, we measure the quality of the partitions of the network using the modularity and Louvain optimisation. We observed that a greater or lesser homogeneity of the magnitudes of the signal transients is related to a higher or lower modularity of the audio-associated visibility network. We also note that these differences are related to musical choices that can establish important differences between musical styles. In this article, we show that the modularity is able to give relevant information to allow the categorisation of 120 musical signs labelled in percussive and symphonic music.

**Electronic supplementary material:**

The online version of this article (doi:10.1007/s41109-017-0052-1) contains supplementary material, which is available to authorized users.

## Introduction

Owing to the need to develop computational resources for the organisation of large digital music libraries, the importance of automatic music classification systems has grown considerably in recent times (Pampalk et al. 2002). Many classification platforms have been proposed (Costa et al. 2012; Ezzaidi and Rouat 2008; Guaus 2009; Panagakis et al. 2009), and despite efforts to find a new path (Goulart 2012; Jennings et al. 2004), most feature extraction tools use knowledge of the audio signal processing field (Eronen 2009; Silla Jr et al. 2006; Tzanetakis and Cook 2002). The descriptors most commonly used in feature extraction are Mel Frequency cepstral coefficients (MFCCs), spectral rollof, spectral flux, zero crossing rate, and low-energy feature. These algorithms carry out their mathematical operations in the time–frequency domain in order to extract three basic characteristics of the musical signal: tone texture (timbre), rhythmic content (time, rhythm, pulse), and tonal content (pitch). In contrast, we propose a way to categorise polyphonic^1^ signals using a topological property of complex networks in this paper, and therefore do not use the same principles traditionally adopted in the analysis of an audio signal. To realise this idea, we first captured the loudness of the audio signal from a calculation of the average intensity of its fluctuations in fixed-size windows (Jennings et al. 2004), creating a series of variance fluctuations of the original signal. After this, we mapped this series onto a graph using the geometrical visibility mapping proposed by (Lacasa et al. 2008). In this mapping, if two points of the series ’see each other’ in the Cartesian plane, an edge is created in the Euclidean plane. Thus, if the visibility of a point in the series is higher, it will have more edges in the graph. At the end of the mapping, the graph inherits the visibility of all local peaks with their respective neighbourhoods within its structure (Lacasa and Toral 2010). Consequently, a variance fluctuation series with few local-but very visible-peaks, will generate graphs with few hubs, with a high degree of connections. On the other hand, a series with many local peaks with a poor visibility will generate graphs with many vertices with a lower level of connections. The analysis of the modularity will identify if the network structure was created from the series with a greater or smaller local visibility.

## Related works

Researchers in the computer music field have used the structural features of complex networks to solve various problems related to music information retrieval, such as musical taste in Internet communities (Buldú et al. 2007), algorithmic composition (Tse et al. 2008), collaborative networks between composers (Park et al. 2015) and music genre classification (Correa et al. 2010). The authors of (Tse et al. 2008) built a network based on a pattern analysis of Bach, Chopin, and Mozart compositions, linking the duration of two notes in a Musical Instrument Digital Interface (MIDI) format that co-occur in a melodic phrase, using universal properties found in these networks to propose rules for algorithmic composition. To analyse the musical tastes of users from their playlists, the authors of (Buldú et al. 2007) used the basic features of networks, where the nodes are the song titles, and edges occur between two song titles, if the title appears in more than one playlist. The authors of (Correa et al. 2010) dealt with music genre classification using the rhythms extracted from MIDI database, transforming it into complex networks. In (Correa et al. 2010), each rhythmic cell is a node, while the sequences of notes define the links between nodes according to a Markov model. The authors of (Jacobson et al. 2008) combined an audio analysis and a network structures to identify communities of artists on the Myspace website, establishing links between two artists who have similar tags on social networks, and audio-based similarity using the MFCCs and entropy. The authors of (Park et al. 2015) studied the topology and evolution of networks of western classical music composers, building links between two composers who co-occur on the same compact disc, linking information about the author, period, and style extracted from the audio file metadata. A characteristic that can be noticed in most scientific papers that use the mapping of complex networks to understand music audio phenomena is the absence of structures formed by links, where the nodes are non-symbolic elements. With the exception of (Jacobson et al. 2008), which used audio data in the network vertex in the first of two phases of the mapping, we have not found another study whose network is formed by the relationship between audio signal points. Considering the survey by (Schedl et al. 2014) that shows various approaches for music content analysis, we also note the lack of methodologies that use complex network properties to perform feature extraction from audio signals. In order to reduce this gap, we propose the modularity of complex networks as a parameter to measure the homogeneity of the onsets of a musical signal in this paper, and with this measure, obtain relevant information that can be used to classify these signals according to a given taxonomy.

## Visibility graphs

Visibility graphs have created bridges between time series analysis and complex network analysis, opening possibilities in the time series field by using a set of new tools. One of these bridges has been used to study long-term correlations, fractal properties, and self-similarity structures (Lacasa et al. 2009; Nunez et al. 2012) that have found applications in temporal observations such as the Nasdaq and *S*&*P*500 daily stock indices (Stephen et al. 2015) and the traffic of information packet series (Andjelković et al. 2015). These studies show that visibility graphs have the ability to capture local trends in time series and measure them through a network analysis. Motivated by these studies, we chose the same type of mapping, seeking to identify how much the persistence of an audio signal time series is associated with the changes in the dynamics influenced by the percussive activity of its musical content. This article will show that the modularity is able to capture the reflections of the self-similarity and the patterns of the persistence of loudness embedded in the network but will not establish a direct relationship with power laws or Hurst exponent calculations, as in (Lacasa et al. 2009).

## Materials and methods

In this section, we first present the database; then, we show the methodological approach to conduct a study of the visibility of an audio signal by using the modularity of complex networks. This methodology was adopted in (Melo et al. 2016). We take a set of 120 audio samples that are 30 s long. Each song is represented by a time series *W*(*i*). In this series, we calculate the subset of variance fluctuations *V*(*j*). For each *V*(*j*), the ‘visibility’ in relation to its successors and predecessors is evaluated, according to the slope comparisons (Lacasa et al. 2008). At the end of the process, the subset *V*(*j*) becomes the graph *G*(*V*(*n*),*V*(*m*)), from which the modularity and the number of communities are estimated.

### Database

In this study we used 120 music files from the Symphonic and Percussive categories, each with 60 songs. For the Symphonic music, pieces by string quartet and full orchestra were selected. The compositions included Bach concertos, Mozart symphonies, and quartets by Debussy, Dutileux, and Ravel. The Percussive category is composed of songs equally taken from six genres with a strong influence and persistent execution of acoustic and electronic percussion instruments: Samba, Forró, Axé, Mangue Beat, Disco, and Trance music. The Samba tracks are songs composed for the celebration of the Rio de Janeiro carnival from 2005 to 2014. In Mangue Beat, there is an influence of electronic pop-rock music mixed with a traditional Afro-Brazilian rhythm called Maracatú. The tracks of Disco music provide a good overview of the musical scene of the 80s. Axé music and Forró are Brazilian rhythms and dances traditionally used in popular festivals such as carnival and rural parties in the northeastern region of the country. Trance represents electronic music with an intense and dancing beat, universally and especially used in youthful entertainment events. The Symphonic and Disco music are chosen from GTZAN^2^ database, and Samba, Axé, Mangue Beat, Trance and Forró tracks are from the author’s personal collection. The Percussive tracks are labelled as Percussive 1 …Percussive 60 (P1–P60), distributed as follows: Disco (P1– P10); Samba (P11–P20); Mangue Beat (P21–P30), Trance (P31–P40), Forró (P41–P50), and Axé (P51–P60). The Symphonic networks are labelled as Symphonic 1 …Symphonic 60 (S1–S60).

### Transforming audio samples in a variance fluctuation series

In this section, we first calculate the variance fluctuations of a musical signal with the same methodology used in (Jennings et al. 2004; Melo 2013). Consider an audio music signal represented by the *W*(*i*) series, with *i*=1⋯*N*. The total number of points *N* is a function *N*=*SR*.*t*, where the sampling rate is *SR*=11,025 Hz and the duration is *t*=30 s. In this work, we delimit *N* at 330,000 points to avoid non-integer values in future calculations. The set *W*(*i*)=*W*(1),⋯,*W*(*N*) is segmented into *m* non-overlapping boxes *λ*= 110 (or 0.01 s). Each box *j*=1⋯*m* is calculated by the standard deviation. In the *j* ^*th*^ box, we have:

1 $$\begin{array}{@{}rcl@{}}  V(j)=\sqrt{\frac{\sum\limits_{(j-1)\lambda+1}^{j\lambda}(W(i)-\overline{W}(j))^{2}}{\lambda-1}}, \end{array} $$

Where the average is given by:

2 $$\begin{array}{@{}rcl@{}}  \overline{W}(j)=\frac{\sum\limits_{(j-1)\lambda+1}^{j\lambda}(W(i))}{\lambda} \end{array} $$

This creates the variance fluctuation subseries *V*(*j*)=*V*1,*V*2,⋯,*Vm*, with 3000 samples.

It is worth noting that the *W*(*i*) signal is calculated with a sampling rate considered to be low for some purposes. However, according to the test done in (Melo et al. 2016), the 11,025 Hz showed better computational performance in the network modelling stage, without loss of information for comparative analysis.

### Transforming variance fluctuations in graphs

Each variance fluctuation point *V*(*j*), *j*=1⋯3000, is considered to be a vertex of the network. To apply the visibility criterion to the series, we will consider each point of *V*(*j*) as an ordered pair (*x*_*j*_,*V*_*j*_), where *x*_*j*_ is the point position in the series. Two vertices (*x*_*a*_,*V*_*a*_) and (*x*_*b*_,*V*_*b*_) are connected if there is a point (*x*_*c*_,*V*_*c*_) between them such that

3 $$\begin{array}{@{}rcl@{}}  \frac{V_{b}-V_{c}}{x_{b}-x_{c}}>\frac{V_{b}-V_{a}}{x_{b}-x_{a}} \end{array} $$

Equation 3 (Lacasa et al. 2008) provides a comparison between the *α*_*bc*_ slope (left side of the equation) and the *α*_*ba*_ slope (right side of the equation). Whenever *α*_*bc*_>*α*_*ba*_, there is visibility between *Va* and *Vb*, and their corresponding nodes are connected in the graph. Otherwise, it does not constitute an edge in the graph. After Eq. 3 is applied to all points of the series following the order *j*=1…3000, we have the visibility of each point of a subset *V*(*j*) mapped onto the graph *G*(*V*(*m*),*V*(*n*)). This means that, from this stage, each song is represented by a visibility graph.

#### Visibility mapping

In this section, we present an example of mapping using visibility graphs to transform an eight-point series into a network.

**Fig. 1:**
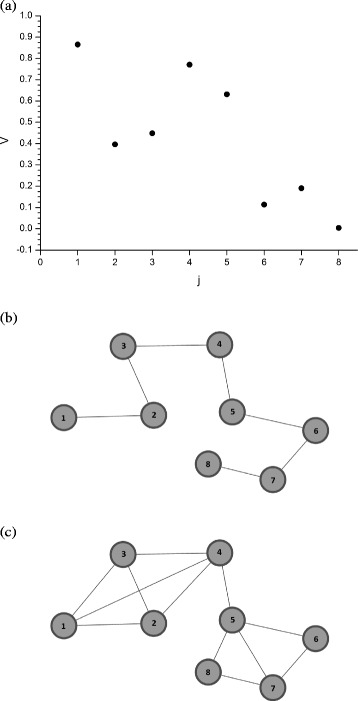
Example of a series transformed into a visibility graph. Source: Author. **a** Series *V* containing eight points **b** Visibility mapping of consecutive points in *V* - Hamiltonian path **c** Visibility mapping of *V*

We utilise the series *V* = {0.865,0.396,0.449,0.770,0.631,0.113,0.190,0.003}, whose representation in the Cartesian plane is given in Fig. 1 a. Each point *j* of the series *V* corresponds to a vertex of the network with the same numbering *j*. The edges between vertices 1 and 2, 2 and 3, 3 and 4, …, 7 and 8 occur because of the trivial visibility between the consecutive points in *V*. This creates a Hamiltonian path (Fig. 1 b).

To determine if there is visibility between non-consecutive points such as *V*1 and *V*4 we calculate the slope value *α*_(1,4)_=−0.032 and compare it with *α*_(2,4)_=0.187 and *α*_(3.4)_=−0.321 according to Eq. 3. We obtain *α*_(1,4)_<*α*_(2,4)_ and *α*_(1,4)_<*α*_(3,4)_, meaning that there is visibility between *V*1 and *V*4, and consequently a connection between vertices 1 and 4 in the network. On the other hand, there is no visibility between points *V*1 and *V*5, since the criterion is not satisfied for the intermediate point *j* = 4 (*α*_(1,5)_>*α*_(4,5)_). As a result, there is no connection between vertices 1 and 5 in the network. Figure 1 c shows the results of the complete mapping of *V*.

### Detecting communities using the modularity

After mapping the variance fluctuations of the audio sample onto a visibility graph, the modularity defined in (Newman 2004) is calculated using as follows:

4 $$\begin{array}{@{}rcl@{}}  Q=\frac{1}{2m}\sum_{(i,j)}\left(A_{ij}-\frac{k_{i} k_{j}}{2m}\right)\delta(c_{i}, c_{j}), \end{array} $$

were *i* and *j* are the nodes of the network, *A*_*ij*_ represents the number of edges between *i* and *j*, *k*_*i*_ and *k*_*j*_ are the sum of the the edges attached to *i* and *j*, *m* is the sum of all edges in the graph, and (*c*_*i*_,*c*_*j*_) is a Kronecker delta function (0 for *c*_*i*_=*c*_*j*_ and 1 for *c*_*i*_≠*c*_*j*_); where *c*_*i*_ and *c*_*j*_ are the communities of the nodes.

The calculation of the modularity can be interpreted as a comparison between the density of local connections and the density of connections taken at random with the same local nodes. The systematic deviations given by Eq. 4 allow us to define the quality of the partition through the quantification of the modularity.

To maximise the modularity efficiently, the Louvain method (Blondel et al. 2008) uses two iterative stages:

- Each node is attributed to its own community. Therefore, the change in the modularity is calculated for each node *i*, removing this node from its own community *C* and moving it to the community of each neighbour *i*. The gain of the modularity is calculated by
  5 $$\begin{array}{@{}rcl@{}}  \Delta Q=\left[\frac{\sum_{in}+k_{i,in}}{2m}-\left(\frac{\sum_{tot}+k_{i}}{2m}\right)^{2}\right]-\left[\frac{\sum_{in}}{2m}-\left(\frac{\sum_{tot}}{2m}\right)^{2}-\left(\frac{k_{i}}{2m}\right)^{2}\right], \end{array} $$
  where $\sum _{in}$ is the sum of the links inside *C*, $\sum _{tot}$ is the sum of the links incident to nodes in *C*, *k*_*i,in*_ is the sum of the links incident to node *i*, *m* is the sum of the links from i to nodes in *C* and *m* is the sum of the weights of all the links in the network.
- Nodes belonging to the same community are grouped together, forming ’supernodes’ of a new network.

These steps are repeated until the maximum modularity is achieved and a community hierarchy is produced.

## Experimental results

### Variance fluctuations of musical audio samples

**Fig. 2:**
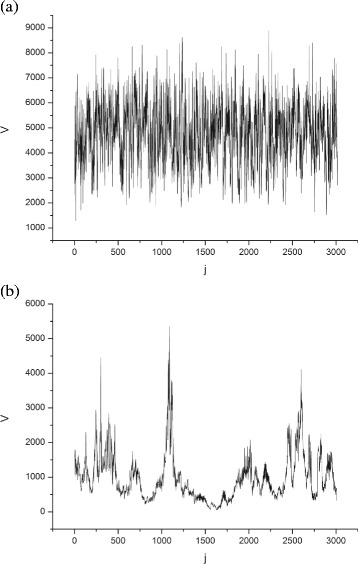
Variance fluctuation series with 3,000 points obtained from 30 s audio files: **a** Unidos da Tijuca’s Samba-Theme - Rio de Janeiro Carnival Party - year 2012 and **b** J. S. Bach - Cantate BWV 156 - Ich Steh Mit Einem Fuss. Source: Author **a** Percussive series **b** Symphonic series

One hundred and twenty variance fluctuation series were calculated by reducing the original signal to 3000 points. Figure 2 illustrates the variance fluctuation series of two audio samples. The first represents the Percussive group and the second the Symphonic group. In Fig. 2 a, we have a numerical series generated from a song with a strong beat generated by a drum set used in Brazilian Samba, and Fig. 2 b shows a portion of a Mozart symphony performed by the string section of an orchestra without percussion instruments. We can notice, by visual inspection, that the first series (percussive series) has a greater homogeneity in relation to the magnitude of the transients than the second series (Symphonic series).

### Audio-associated visibility networks

**Fig. 3:**
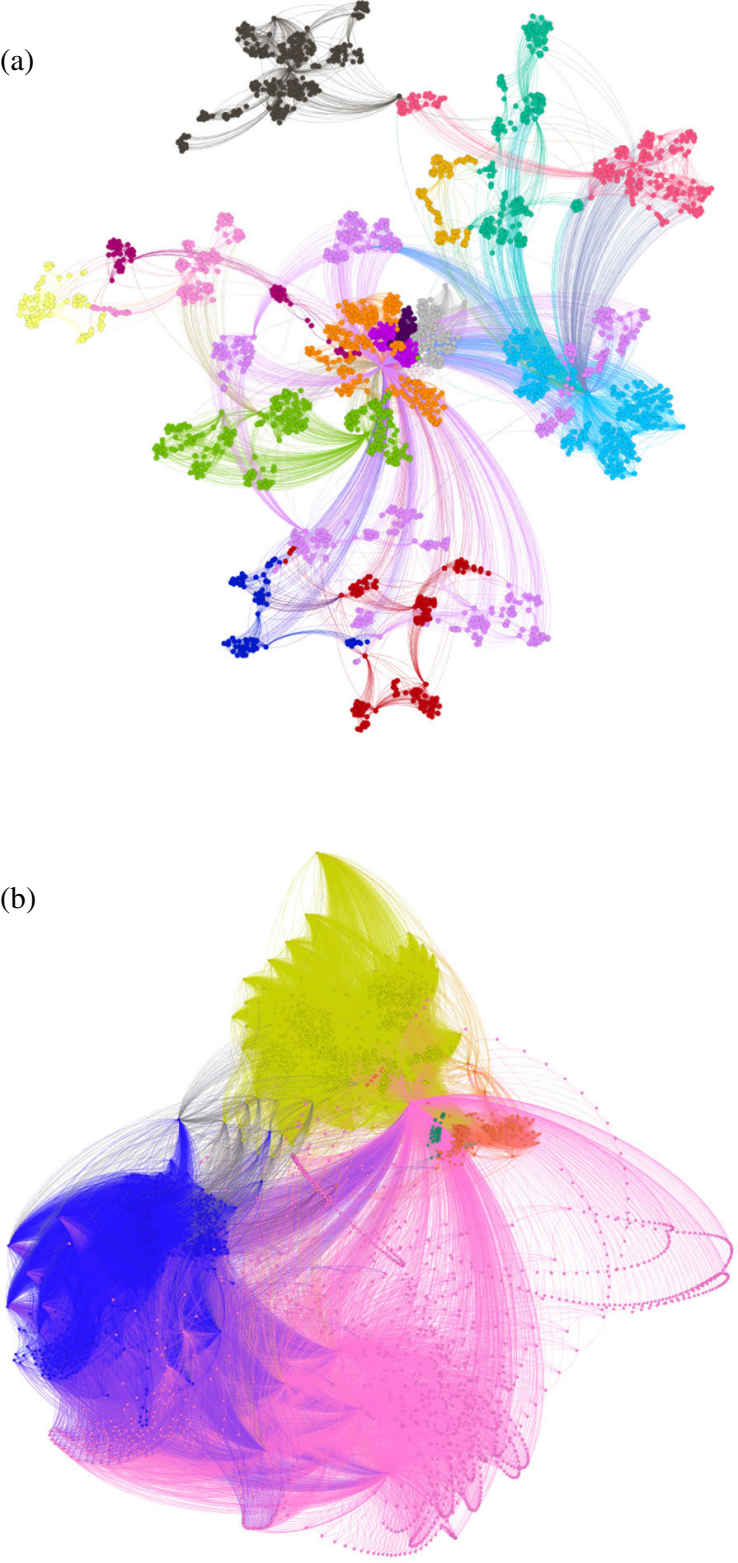
Audio–Associated Visibility Networks of the series in Fig. 2 a and b. The colours represent the modularity classes of each network. Q is the modularity, and Nc is the number of communities. Source: Author. **a** Percussive network (Q=0.790, Nc=17) **b** Symphonic network (Q=0.551, Nc=5)

We mapped 120 variance fluctuation series into visibility networks: 60 Symphonic and 60 Percussive networks. Figure 3 shows two of these audio-associated visibility networks. Figure 3 a shows the mapping of a Samba song, whose series of variance fluctuations is shown in Fig. 2 a. The second (Fig. 3 b) is an audio-associated visibility network of the J. S. Bach BWV 156 Cantata, shown in Fig. 2 b. In these two representations, the modularity classes appear in different colours, indicating the communities formed by each network. In the Percussive network (Samba), the qualitative (Q) and quantitative (Nc) superiorities of the communities in relation to the Symphonic network is quite remarkable. “Modularity results” section will present the overall results that allows inference of the trends presented by each group based on a comparison of the Q and Nc values of the Percussive and Symphonic network sets.

The average number of links of the Symphonic and Percussive networks are, respectively, 67,754±19,238 and 22,426±4,130. The results show a significant difference between the mean values of the links generated between the two types of networks. Considering that series with a great homogeneity of onsets (Fig. 2 a) have a lower local visibility than series with low homogeneity of onsets (Fig. 2 b), and that Percussive series tend to have a greater homogeneity, and therefore lower local visibility than the Symphonic series, we can infer that the total number of links has a direct relation with the visibility of the predominant local peaks. This indicates that, on average, Symphonic series have greater local visibility than Percussive series.

### Modularity results

#### Q values

**Fig. 4:**
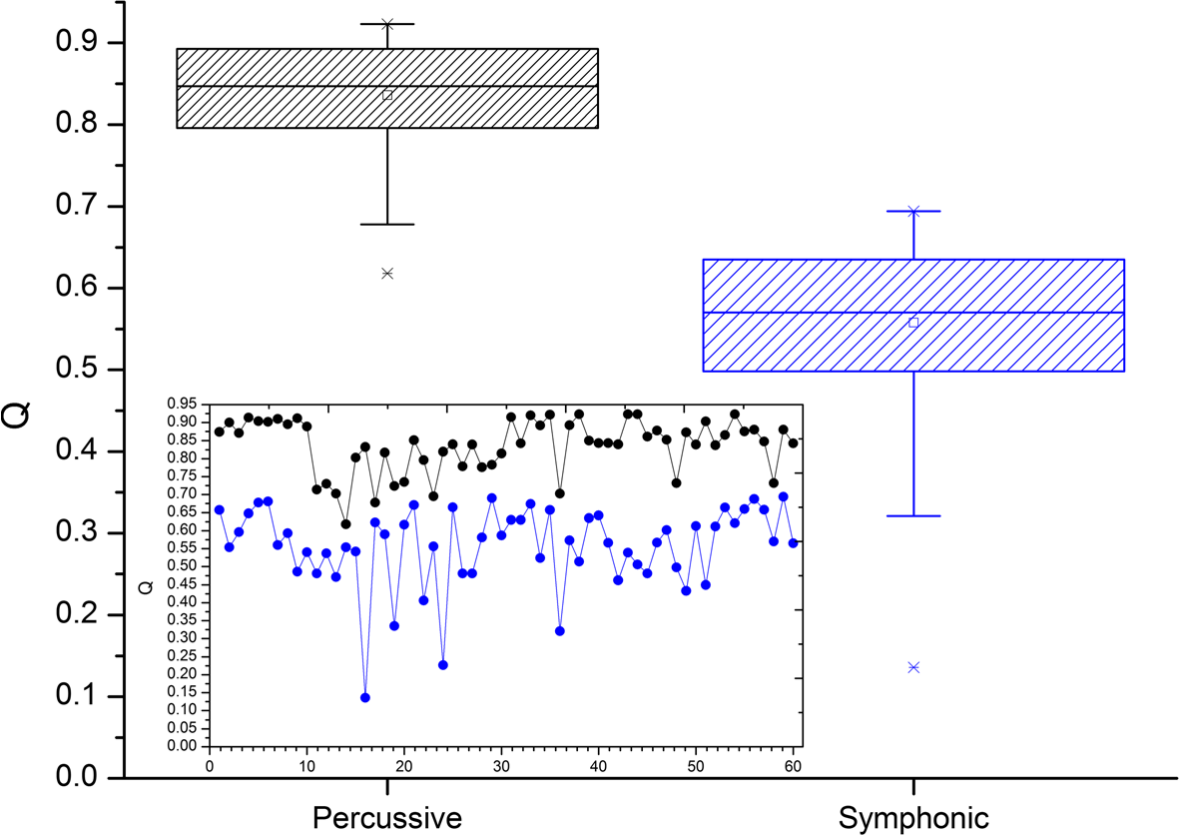
Modularity of 60 Percussive (boxplot and dots in black) and Symphonic (boxplot and dots in blue) visibility networks. Source: Author

Figure 4 shows boxplots and the values of the modularity for each network of the two categories (inset). The average modularity of the Percussive and Symphonic networks are respectively <*Q*>=0.836±0.074 and <*Q*>=0.558±0.111. The Q values of each network are indicated with the numbers 1–60. The Symphonic networks exhibited a less compact modularity distribution than the Percussive networks, with a coefficient of variation of 19.9*%* against 7.4*%* of the Percussive Networks. The analysis of variance (ANOVA) comparison test approved, with 95% confidence, the hypothesis of the significant difference between the average modularities of the two groups. The smallest Q value was *Q*=0.136 for the visibility network S16 (Mozart’s Symphony 39 in E flat Major - k 54), and the highest modularity (*Q*=0.923) was achieved by four Percussive networks of Trance (P38), Forró (P43, P44), and Axé (P54).

**Fig. 5:**
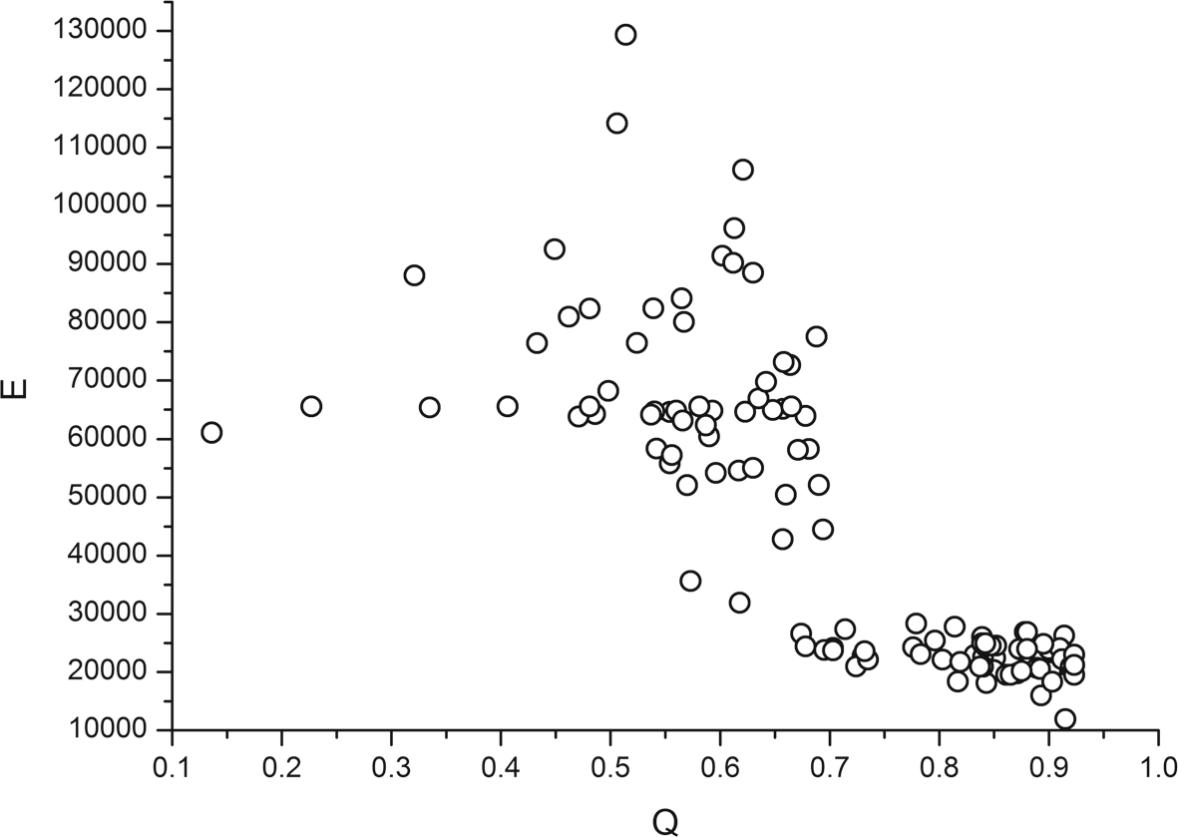
Dispersion of the modularity (Q) as a function of the number of edges (E) of their respective visibility networks. Source: Author

Using the dispersion graph of the modularity (Q) as a function of the number of edges (E) shown in Fig. 5, we can infer a correlation with a negative tendency, which in fact is confirmed by calculating the value of the Pearson coefficient k = -0.78. This means that for most data, a greater modularity means that there are fewer edges in the network. At the same time, according to the results in “Audio-associated visibility networks” section, Percussive networks tend to have higher modularities and fewer edges, unlike Symphonic networks. This indicates that in Fig. 5, the Percussive networks occupy the rightmost part along the Q axis and the Symphonic networks the leftmost part. Machine learning with the J45 decision tree (of the same family as the renowned C4.5) establishes that above *Q*=0.694, networks are classified as Percussive and below this value as Symphonic. With this criterion, the classification algorithm achieved correctly classified instances at a rate of 97.5*%*, with only two Percussive networks below and one Symphonic above the Q limit used as a rule by the decision algorithm. The same training was done using E as classification attribute, and the percentage correct was very close to that achieved by the modularity.

#### Number of communities

Globally, the number of network communities follows the same feature found in the calculation of the modularity; there exists a very clear distinction between the two classes, where the Percussive networks outweigh the Symphonic networks for most Nc values. The average values obtained were <*Nc*>=19.0±5.1 for Percussive, and <*Nc*>=8.5±2.1 for Symphonic networks.

#### A closer look at some percussive and symphonic networks

From Fig. 4, we can see that some points stand out from the rest of the group because they have reached discrepant or extreme values. In the following, we will discuss the possible causes of this behavior, behaviour, combining musical and statistics similarities.

**Fig. 6:**
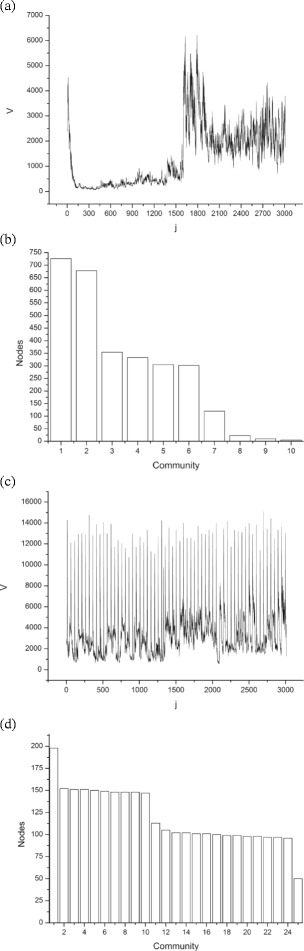
Variance fluctuations of two audio samples and their respective distributions of nodes per community, obtained from the visibility networks created by mapping these fluctuations. Source: Author. **a** Variance fluctuation series of Symphonic track 24 (Mozart’s Symphony 41 (k 551)). **b** Distribution of nodes per community of the Symphonic track 24 visibility network. **c** Variance fluctuation series of Percussive track 4 (song “Run Back” by Carl Douglas). **d** Distribution of nodes per community of the Percussive track 4 visibility network
- *Networks S16, S19, and S24* - These networks have a modularity with very low values (0.141, 0.227, and 0.355). The audio excerpts associated with these networks also have a common musical feature. In all of them, there is a sudden change in the dynamics, strongly influenced by the presence or absence of a timpani^3^. It created a particular topology in the variance fluctuations of these audio signals with great ’valleys’ followed by high ’peaks’ favoring visibility graphs with large hubs, and nodes per community distributions with a very low number of nodes in some communities, and very hight nodes in few others, forming few clusters and a low modularity (Fig. 6 b).

- *Networks P1-P10* (Fig. 5 - first 10 white dots) - They achieved a greater magnitude and shorter variance in modularity (<*Q*>=0.897±0.015) compared to P11-P60 (<*Q*>=0.767±0.064)) (thirty last white dots) and Symphonic networks (black dots). Looking at the distributions of vertices per community, of all networks, we observed higher homogeneity for the P1-P10 distributions. Figure 6 d shows one of these distributions. Musically, the P1-P10 networks represent songs of the 80s, which is characterised by the same danceable groove in every song. This may have influenced the results of the modularity, and the distribution of degrees per community.

### Studying the homogeneity of variance fluctuations

In this section, we will show the results of the distribution of nodes per community. This distribution will help us to understand the relationship between the homogeneity of the variance fluctuations and the musical characteristics of the audio track. In fact, variance fluctuations are a significant representation of the main transients of the audio signal. The transients that have the greatest magnitude (peaks) are directly related to the attack of the notes of the instruments present in the polyphonic signal. These attacks mark the most characteristic times in the music and are also present in the sudden changes in the dynamics^4^.

Figure 5 a and c show the variance fluctuations of two musical pieces, and Fig. 6 b and d show respective distributions of nodes per community, calculated from their associated visibility networks. Figure 6 a shows an excerpt from Mozart Symphony 41 (k 551), where two abrupt changes in the dynamics can be noted: the first occurs at the end of the first initial 1.2 s (*j* = 0 to *j* = 120), and the second at 16 s (*j* = 1603). In this first stretch, the orchestra performs a tutti in *fortissimo* and soon follows in *pianissimo*, from 1.2 to 16 s (*j* = 120-1603). From 16 to 30 s (*j* = 120-3000), the orchestra changes again to fortíssimo and remains in this way until the end. Owing to this abrupt change in the visibility network associated with this signal, *Q*=0.227. We can also observe that in the distribution of nodes per community, about 50% of the nodes belong to only two communities, and three of the ten communities have less than 2.5*%* of the total of nodes. This imbalance in the distribution is a reflection of the abrupt change that has determined a great valley within the signal. In this way, the mapping by the visibility created a network where the influence of the two great peaks near the created valley was determinant in the creation of the communities, since they assumed the role of network hubs with a very high level of connections, which resulted in a network with two communities with a very high density of connections.

The distribution of nodes per community in Fig. 6 d shows a situation different from the previous view. In this case, this distribution represents disco music, where the bass, voice and percussion instruments, mark the strong tempos of the music with a high homogeneity, producing peaks associated to the onsets that favour the creation of visibility graphs associated with many communities with the same number of nodes, and a fairly homogeneous general distribution. At the end this signal has a visibility network with a modularity *Q*=0.915, indicating that within the formed communities, the density of the intrinsic connections to the communities is much greater than the density of the connections taken randomly.

### Results of other network properties

In addition to the modularity (Q) and the number of communities (Nc) presented in Sections Q values and Number of communities, we also calculated the average degree (〈*k*〉) and the clustering coefficient (Clust). Table 1 summarises the mean values of these properties.

**Table 1:** Average of topological properties of 60 percussive and symphonic networks

	Q	Nc	〈*k*〉	Clust
Percussive net	0.836 ± 0.074	19.0 ± 5.0	15.12 ± 2.37	0.838 ± 0.056
Symphonic net	0.558 ± 0.111	8.5 ± 2.1	46.27 ± 11.53	0.841 ± 0.070

Table 1 indicates that the two types of networks reached a clustering coefficient (Clust) greater than 80%, indicating that in both cases, the mapping using the visibility criterion favours the creation of networks with a high triangulation rate. The Percussive networks exhibited mean values of D and 〈*k*〉 that are significantly lower than those of the Symphonic networks, suggesting an inverse tendency to that presented for Q and Nc. In summary: percussive networks show a tendency for high modularity and number of communities, and a low average degree, whereas Symphonic networks tend to exhibit a low modularity and number of communities and a high average degree. This behaviour can be explained as follows. Since Symphonic networks tend to display more signals with large magnitude peaks followed by peaks of a smaller magnitude than the Percussive networks (Fig. 2 a and b), their visibility networks also tend to have more vertices with a very high degree. This makes Symphonic networks prone to having the upper average degree than Percussive networks. This will impact the global connectivity for both types of networks. As a result, Symphonic networks tend to have more hubs, a high local connectivity, and a low modularity, unlike Percussive networks (Fig. 3 a and b). A comparison of the modularity with the other properties reinforces the idea that the musical choices found in the musical sections labelled as Percussive tend to exhibit signals with a greater homogeneity than those in Symphonic networks according to the discussion in Section Studying the homogeneity of variance fluctuations.

## Conclusion and future work

In this article, we transform 120 stretches of audio into visibility graphs and calculate the modularity of these networks. We realise that a greater or lesser homogeneity of a given polyphonic signal is related to higher or lower modularity of the his visibility network. After observing the descriptive statistics of the Q values and the results of the supervised categorisation of the decision tree, we conclude that the modularity is able to give relevant information for pattern recognition and classification of musical signals according to the taxonomy used in the experiment presented in this paper. These results also lead us to conclude that Percussive songs are associated with high values of the modularity, unlike Symphonic songs, and that these tendencies are strongly linked to the musical choices that influence the design of signal transients. Specifically, we found that Symphonic songs use much more variations in the dynamics and less rhythmic persistence than Percussive songs, resulting in more heterogeneous signals and visibility graphs with lower Q values. We also found that owing to musical choices, signals from different categories may have the modularity of their networks within the range of modularity inherent to the others. This justifies the presence of the rare overlappings and outliers observed in this experiment. In future work, we will perform a comparative study of several music genres with a large audio database, evaluating the classification performance of the properties of complex networks in relation to several algorithms in the audio signal processing field. We also intend to apply other optimisation methods to the modularity in the detection of communities in audio-assiciated visibility graphs and to compare the results with the Louvain method (Additional file 1).

## Endnotes

^1^ A polyphonic signal is a signal that has more than one voice or sound.

^2^ GTZAN Genre Collection is a database widely used in musical information retrieval research. It was proposed by 8 and is available at http://marsyasweb.appspot.com/download/data_sets/.

^3^ A set of two or three large drums (called kettledrums) that are played by one performer in an orchestra http://www.merriam-webster.com/dictionary/timpani.

^4^ Changes in loudness in a piece of music http://dictionary.cambridge.org/dictionary/english/dynamicsor the varying levels of volume of sound in different parts of a musical performance https://en.oxforddictionaries.com/definition/dynamics.

## Additional file

Additional file 1 Additional information. (PDF 451 kb)
