# Supplementary material for: Categorisation of polyphonic musical signals by using modularity community detection in audio-associated visibility network
Source: Appl Netw Sci. 2017 Oct 10;2(1):32. doi: 10.1007/s41109-017-0052-1 (PMC6214251; doi:10.1007/s41109-017-0052-1)
Supplement: Supplementary file 1 — Additional information. (PDF 451 kb) [file 41109_2017_52_MOESM1_ESM.pdf]

Additional information

Topic #1. Motivation and Loss of data quality with downsampling.

In our article we distinguish percussive influence on two categories of musical audio signals through the homogeneity of their onsets. In the methodology we adopt this homogeneity is represented by the fluctuations of signal variance and is quantified through the modularity of visibility graphs mapped from these fluctuations. The results below show that the reduction of points by downsampling does not influence the characterization of the homogeneity of the signals nor prejudice the comparative analysis used to distinguish the clusters through the modularity of the visibility networks. Figures 1a and 1c show the same audio signal with different sampling rates and their respective variance fluctuations calculated with different box sizes (Figures 1b and 1d). We can note that the downsampling did not cause distortions in the perception of homogeneity of the signals expressed in their respective series of variance.

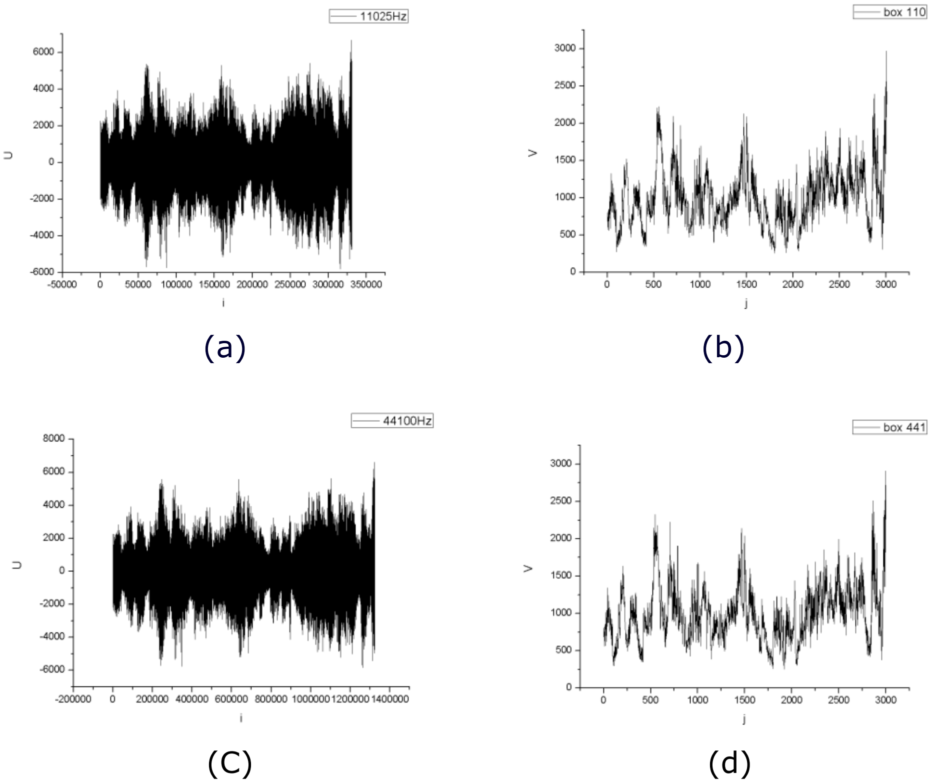

(a) (b)

(C) (d)

Fig. 1

Through Figure 2 we can see how the inherent homogeneities of percussive and symphonic signals can be easily distinguished through their respective series of variance calculated with λ=110 and sampling rate 11.025 Hz. The modularity of the visibility networks mapped from these variance fluctuations is the reflection of the inherent homogeneity of each musical category.

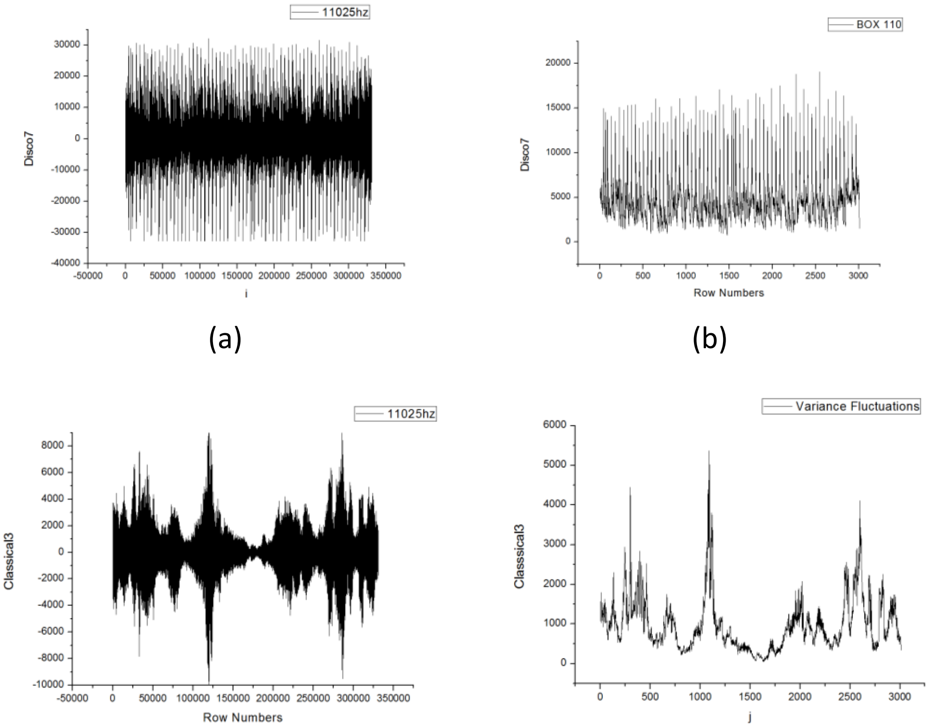

(a) (b)

(c) (d)

Fig. 2

Table 1, Figures 3 and 4 show that the modularity of each group does not change significantly with decreasing sampling rates, preserving the overall behavior in the comparative analysis between the two groups.

Table 1

	Sample Rate	Box Size	<Q>
Percussive	11025Hz	110	0.816±0.082
	44100Hz	441	0.865±0.048
Symphonic	11025Hz	110	0.540±0.129
	44100Hz	441	0.573±0.108

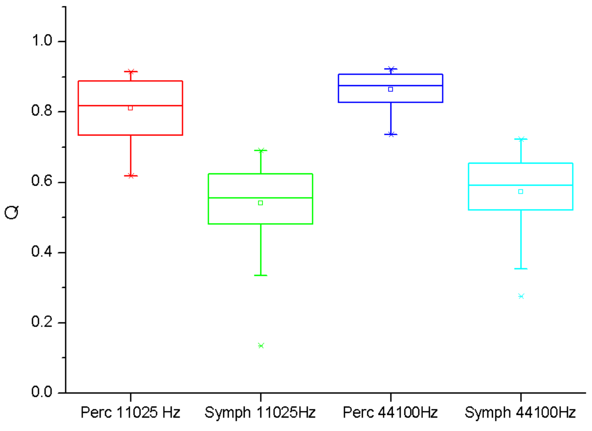

1.0
0.8
0.6
0.4
0.2
0.0
Q
Perc 11025 Hz
Symph 11025Hz
Perc 44100Hz
Symph 44100Hz

Fig. 3

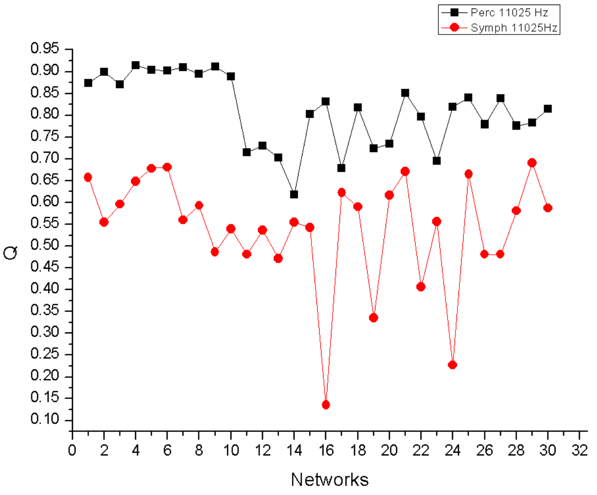

Perc 11025 Hz
Symph 11025Hz
Q
Networks

(a)

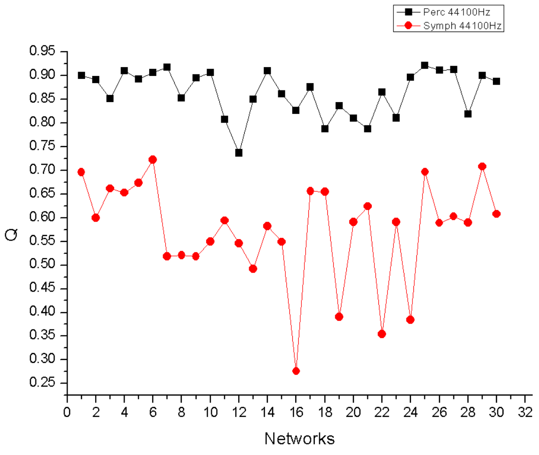

Perc 44100Hz
Symph 44100Hz
Q
Networks

(b)

Fig. 4

Figure 5 shows the results of the Tuckey (ANOVA) test for the analysis of variances for the modularity of the Symphonic (S) and Percussive (P) visibility networks. This test shows with 95% confidence that there are no significant differences between the average modularities of the networks of the same musical category and with different sampling rates (white boxes), but that there are significant differences between independent average modularities of different categories (black boxes) Of the sampling rate. On the other hand, significant differences are perceived between different categories.

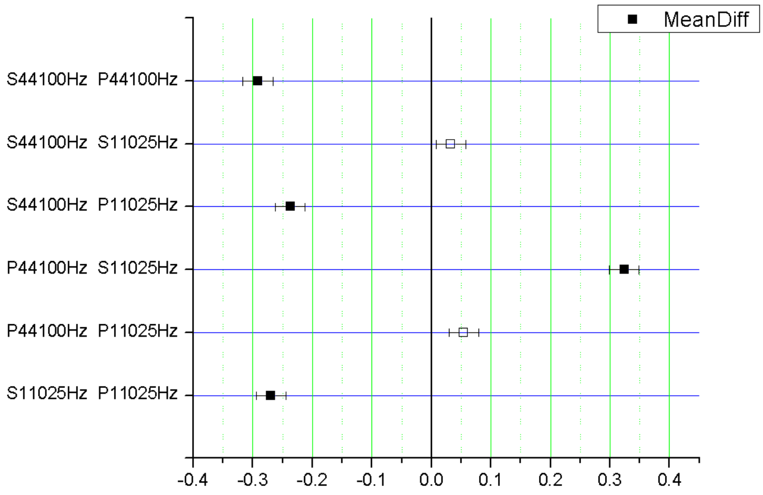

Fig. 5

These results indicate that the choice of sampling rate of 11.025Hz does not imply loss of data quality.
